# Ovarian cancer cell lines derived from non-serous carcinomas migrate and invade more aggressively than those derived from high-grade serous carcinomas

**DOI:** 10.1038/s41598-019-41941-4

**Published:** 2019-04-02

**Authors:** Amelia Hallas-Potts, John C. Dawson, C. Simon Herrington

**Affiliations:** Edinburgh Cancer Research Centre, Institute of Genetics and Molecular Medicine, University of Edinburgh, Crewe Road South, Edinburgh, EH4 2XR UK

## Abstract

The term ovarian cancer describes a heterogeneous group of tumours that grow in the ovary but are not necessarily of ovarian origin. Recent genomic analysis has shown that many of the most commonly used ovarian cancer cell lines have been mischaracterised, leading to erroneous conclusions and a gap in the translation of laboratory research into novel treatments for patients. Here, we use 10 epithelial ovarian cancer cell lines to investigate 2D migration, cell cycle parameters and 3D invasion behaviour into different substrates and find significant differences between the behaviours of cell lines from different origins. Cell lines derived from non-serous carcinomas migrated more quickly and were more likely to invade into Matrigel and collagen I substrates than cell lines derived from high-grade serous carcinomas. However not all cell lines derived from non-serous carcinomas exhibited similar invasive behaviour. These findings may reflect differences in the behaviour of the primary tumour types from which the cell lines were derived, given that high-grade serous carcinomas typically expand and spread over peritoneal surfaces. These results provide the basis of an *in vitro* model for identifying differences between ovarian cancer tumour types.

## Introduction

Ovarian cancer is the most lethal gynaecological disease in women and, in 2018, there will be approximately 300,000 new diagnoses of ovarian cancer worldwide^1^. Despite the improvement in prognosis for most solid tumours, epithelial ovarian cancer prognosis remains relatively unchanged^2^, with only one major new treatment introduced in the last 30 years^3^. The term ovarian cancer describes several histotypes which show distinct cellular origin, molecular aberrations and disease progression in patients^4^; however, this heterogeneity is not reflected in the treatments currently available. Furthermore, the term ovarian cancer may be misleading as many ovarian cancers arise from non-ovarian tissue. The single consolidating feature of ovarian cancers is the localised dissemination of tumour cells to the ovary and pelvic organs^2^. Ovarian cancer is difficult to identify clinically since patients often present with non-specific symptoms such as bloating and fatigue^5^. Indeed, 80% of women are diagnosed at an advanced stage where 5-year survival is only 5% compared to 92% if the cancer is detected early^5^. For women diagnosed at a late stage, the treatment options remain limited and, even if the cancer is detected early, the chance of relapse is 70% within 18 months^5^.

**Figure 1:**
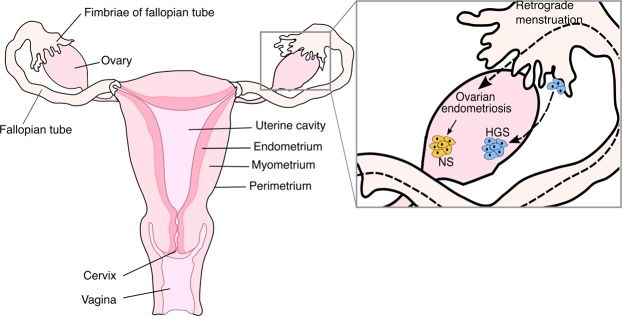
Anatomy of the female reproductive system and cancer biology of the ovary. The inset depicts models of high-grade serous (HGS, blue) and non-serous (NS, yellow) ovarian cancer pathogenesis. Most HGS ovarian carcinomas arise in the fallopian tube fimbria and implant on the ovary whereas most NS ovarian carcinomas arise from ovarian endometriosis which is thought to occur from retrograde menstruation. Dashed lines depict processes (retrograde menstruation or implantation of HGS cells), solid lines depict the progression of disease (ovarian endometriosis to NS carcinoma).

Epithelial ovarian cancer accounts for 90% of all ovarian cancer diagnoses^6^; it encompasses all cancers that arise from epithelium and involve the ovary^7^. The most common histological types are high-grade serous, low-grade serous, endometrioid, clear cell and mucinous carcinomas. High-grade serous ovarian carcinomas (HGS) commonly arise in the epithelium of the fallopian tube fimbria and subsequently present as apparently ovarian tumours after implantation in the ovary (Fig. 1)^7^. These tumours present at an advanced stage, are fast growing and spread throughout the peritoneal cavity^8^. Endometrioid and clear cell carcinomas are non-serous tumours (NS) and have strong links to endometriosis, a disease that affects 10% of the female population^9^. Ovarian endometriosis is thought to arise from retrograde menstruation and results in endometrial tissue growing outside the uterine body, most commonly on the ovaries (Fig. 1)^7^. NS tumours generally present at an early stage where they have not spread beyond the ovary but have formed a large tumour mass. Non-serous tumours have been shown to be mutationally distinct from serous ovarian tumours^10^ but the differences in invasive behaviour of these tumours are poorly described.

In 2013 Domcke *et al*.^11^ found that many of the most commonly used ovarian cancer cell lines do not genetically resemble the tumour types they have been used to investigate. This was confirmed on a different but overlapping panel of cell lines in 2014 by Beaufort *et al*.^12^. These findings highlight a new challenge for ovarian cancer research as many previous findings are now misleading and researchers must re-evaluate their choice of cell lines. Therefore our study focuses on distinguishing the behaviour of ovarian cancer cell lines from different origins to determine whether cell lines with the same genomic characterisation show similar behaviours. We use two panels of five cell lines characterised by Domcke *et al*.^11^ and Beaufort *et al*.^12^; one panel represents high-grade serous carcinoma and the other non-serous carcinoma (endometrioid and clear cell). We show clear behavioural differences between high-grade serous and non-serous ovarian cancer cell lines and propose the use of these cell lines as an *in vitro* model to investigate distinct ovarian cancer tumour types.

## Results

### Criteria for the cell line panel

The panel of cell lines was categorised based on the genomic profiles outlined by Domcke *et al*.^11^ and Beaufort *et al*.^12^. On this basis, OVCAR3, COV318, PE01, PE04 and FUOV1 were considered high -grade serous (HGS) and A2780, OVCAR8, OAW42, SKOV3 and TOV21G non-serous (NS). The keratinocyte-derived HACAT cell line was included as a non-ovarian control.

### NS cell lines migrate faster than HGS cell lines

**Figure 2:**
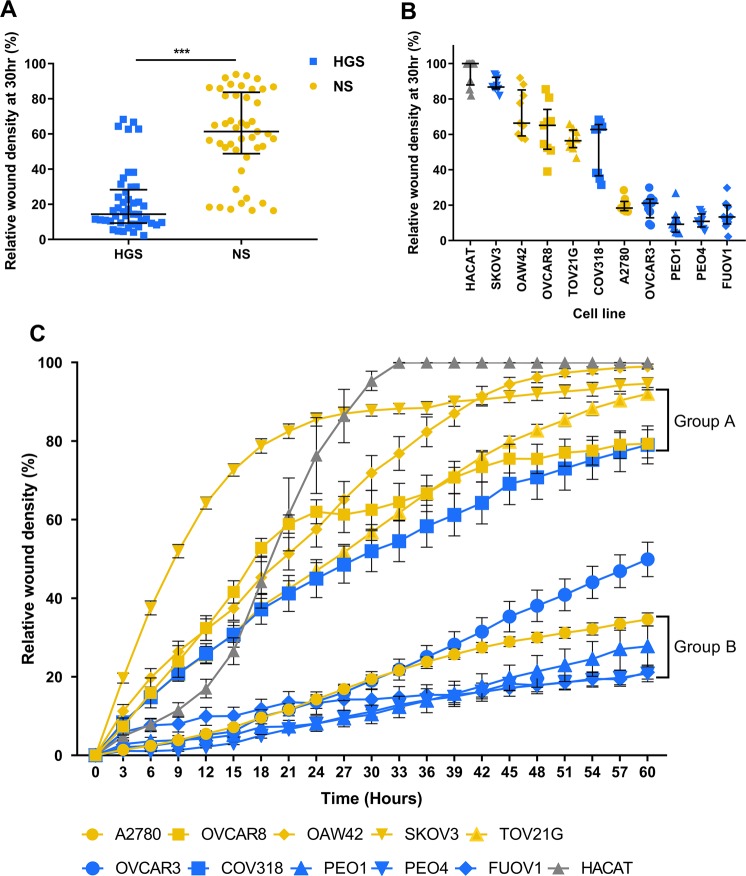
Wound healing behaviour. (**A**). Relative wound density at 30 hr for high-grade serous (HGS) cell lines compared to non-serous (NS) cell lines (median ± IQR). HGS median = 14.36 ± 19.03, NS median = 61.38 ± 34.9 Mann-Whitney U test, p < 0.001, U = 1795. (**B**). Relative wound density at 30 hr for each cell line (median ± IQR). (**C)**. Relative wound density over 60 hours (mean ± SEM). Two-way repeated measures ANOVA identifies two populations of cell lines (p < 0.001). Group A cell lines (COV318, TOV21G, OVCAR8) show no significant difference between relative wound density at >75% of time points (p < 0.005). Group B cell lines (A2780, PEO1, PEO4, FUOV1) show no significant difference between relative wound density at all time points (p < 0.005). (HGS: blue, NS: yellow, Control: grey). Experiments were carried out in triplicate on 3 separate occasions for each cell line. ***p < 0.001.

2D migration behaviour was assessed by relative wound density using the IncuCyte ZOOM® wound healing assay (Fig. 2).

Statistical analysis at 30 hours showed that the relative wound density of NS cells was significantly higher than HGS cells (Fig. 2A, Mann-Whitney U test p < 0.001), highlighting that NS cell lines migrate faster than HGS to close the wound. Statistically, the HGS and NS panels of cell lines show distinct migration behaviour when considered as groups. However, analysis of the behaviour of individual cell lines shows variability between lines (Fig. 2B). In particular, COV318 and A2780 cells show different migration behaviour from the other cells in the HGS and NS panels respectively. COV318 migrates faster than the other HGS cell lines, behaving more similarly to NS cells; whereas A2780 migrates slower than the other NS cell lines, behaving more similarly to the HGS panel. This is evident for wound density at all time points over the 60 hours (Fig. 2C).

Two-way repeated measures ANOVA was used to further investigate differences between cell line behaviour over time, identifying statistically significant differences between the cell lines (Fig. 2C, p < 0.001). Comparison of the behaviour of each cell line at each time point using Tukey’s multiple comparisons showed that HACAT, SKOV3, OAW42 and OVCAR3 behave significantly differently from each other and all other cell lines at >75% of time points. However, the other cell lines divided into two groups, A and B (Fig. 2C). Group A is composed of the cell lines OVCAR8, TOV21G and COV318, which show no significant difference in behaviour from each other at >75% of time points. Group B is composed of the cell lines A2780, PEO1, PEO4 and FUOV1 which show no significant difference in behaviour from each other at all time points. Estimation of cell line doubling times by cell counting showed no systematic relationship between doubling time and migration behaviour: A2780 14 hr, HACAT 15 hr, TOV21G 18 hr, OAW42 25 hr, SKOV3 36 hr, OVCAR3 47 hr, OVCAR8 54 hr, PEO1 82 hr, PEO4 94 hr, COV318 75 hr, FUOV1 98 hr and see Fig. 2.

### Fewer HGS than NS cell lines are in G_0_/G_1_ phase of the cell cycle

**Figure 3:**
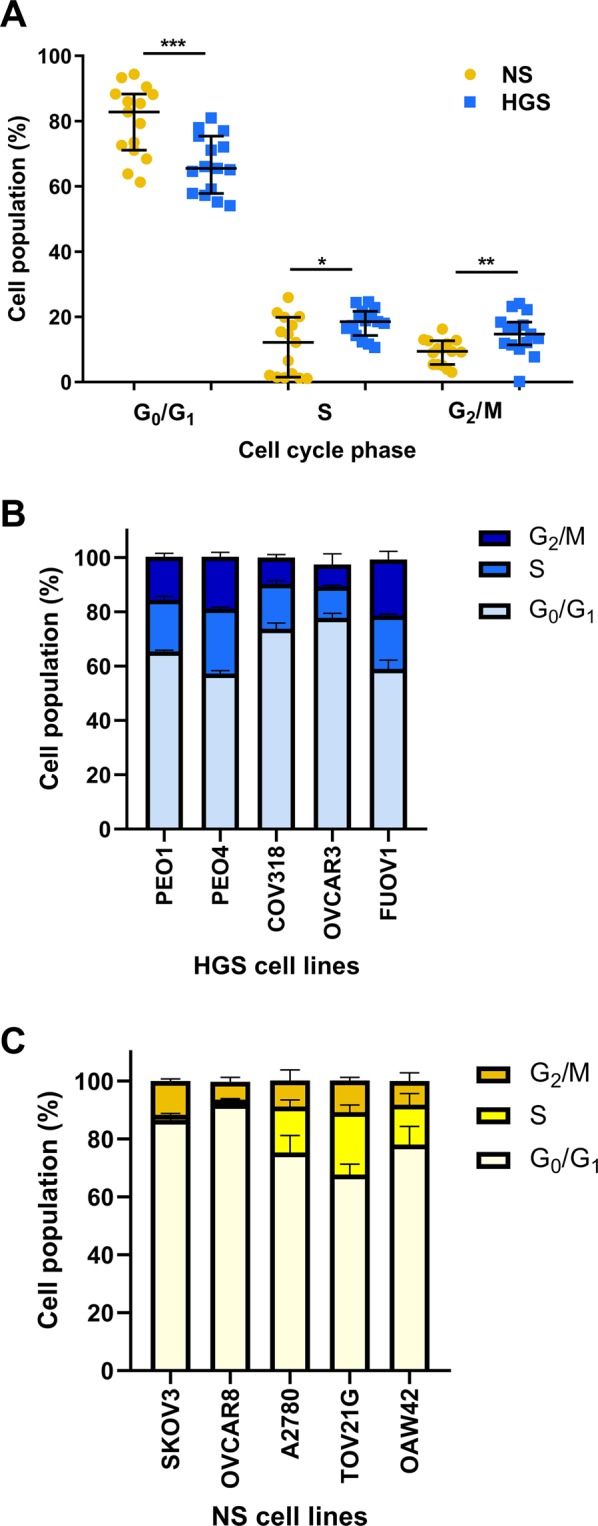
Characterisation of cell cycle behaviour. (**A**). The percentage of cells at each phase of the cell cycle for non-serous (NS) compared to high-grade serous (HGS) cell lines (median ± IQR). *G*_*0*_*/G*_*1*_: HGS median = 65.5 ± 17.6; NS median = 82.8 ± 17.2 Independent samples t-test, equal variance p = 0.001, t = −3.70, df = 28. *S:* HGS median = 18.5 ± 7.4; NS median = 12.2 ± 18.4. Mann Whitney U test, p = 0.033, U = 61.5 *G*_2_*/M:* HGS median = 14.7 ± 7; NS median = 9.4 ± 7.3. Independent samples t-test, equal variance p = 0.008, t = 2.879, df = 28. (**B**). The percentage of cells at each phase of the cell cycle for HGS cell lines (mean ± SEM). **C**. The percentage of cells at each phase of the cell cycle for NS cell lines (mean ± SEM). Experiments were carried out in triplicate on 3 separate occasions for each line.

Flow cytometry was used to determine the cell cycle behaviour of the panels of cell lines at 80% confluence. The percentages of cells in G_0_/G_1_, S and G_2_/M phase were quantified. Significant differences were found between HGS and NS cell lines at each phase of the cell cycle, with significantly fewer HGS cells in G_0_/G_1_ phase compared to NS cells (G_1_/G_0_: t-test: p = 0.001 Fig. 3A). Conversely, significantly more of the HGS cell population was in S and G_2_/M phases compared to NS cell lines (S: Mann-Whitney U test p = 0.033. G_2_/M: t-test: p = 0.008, Fig. 3A). Notably, however, although HGS cell line cell cycle behaviour is significantly different from NS cell lines overall, there is overlap between the cell line groups (Fig. 3B,C).

### NS cell lines are more likely to invade than HGS cell lines

**Figure 4:**
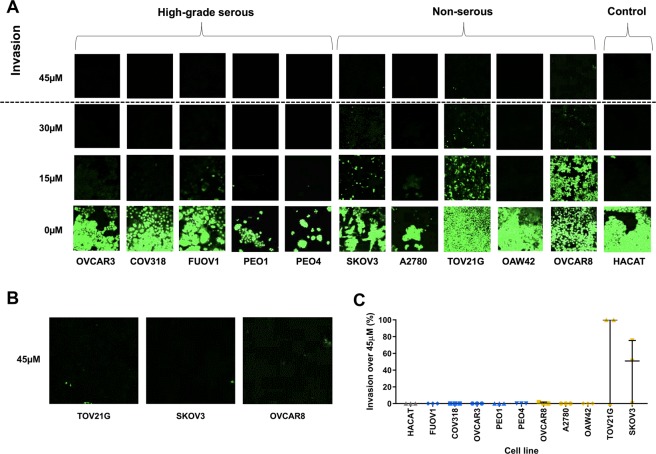
Invasion into Matrigel. (**A**). Representative z-step confocal microscopy images taken at 15 µm intervals through the Matrigel for each cell line. Cells were stained with SYTO™9 green fluorescent nucleic acid stain. (**B**). Larger representative images from A showing TOV21G, SKOV3 and OVCAR8 invasion over 45 µM. (**C**). Invasion above 45 µm for each cell line (median ± IQR), normalised to the most invasive cell line. Experiments were carried out on 3 separate occasions for each cell line; 4 images were captured per repeat per cell line.

The inverted transwell invasion assay was used to determine the invasive potential of HGS and NS cell lines into Matrigel. As described previously^13^ cells that invade above 45 µm into the matrigel were considered invasive; cells below 45 µM are not considered invasive as it is likely they have only migrated across the transwell membrane. By these criteria, only 3 of the 11 cell lines investigated showed evidence of invasion, all of them NS cell lines (Fig. 4). TOV21G and SKOV3 were the most invasive cell lines; OVCAR8 showed only a very small amount of invasion; no other cell line invaded above 45 µm in any experiment.

### Invasive behaviour of ovarian cancer cell lines differs in collagen matrix compared to Matrigel

**Figure 5:**
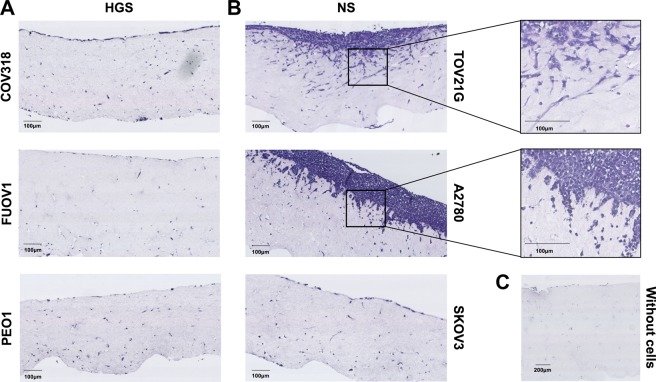
Organotypic invasion assay. Representative images of haematoxylin and eosin (H&E) stained sections showing cell lines cultured on top of collagen/fibroblast matrices at day 10 of the organotypic invasion assay. Images captured using Hamamatsu Nanozoomer XR at 40X objective magnification. (**A**). High-grade serous (HGS) cell lines COV318, FUOV1 and PEO1. (**B**). Non-serous (NS) cell lines TOV21G, A2780 and SKOV3. TOV21G invaded to a maximum depth of 250 µm. A2780 invaded to a maximum depth of 150 µm. (**C**). Collagen/fibroblast matrix with no cell lines cultured on top. Experiments were carried out on 3 separate occasions for each cell line.

This experiment was carried out on the 2 most invasive cell lines (TOV21G and SKOV3), the 2 least migratory cell lines (FUOV1 and PEO1) and the 2 cell lines that behaved differently from the other cell lines in the HGS and NS groups in the migration assay (A2780 and COV318). In the collagen invasion assay, only 2 cell lines invaded into the collagen matrix, both NS cell lines (TOV21G and A2780). None of the HGS cell lines proliferated on top of or invaded into the collagen matrix (Fig. 5). SKOV3 showed prominent invasive behaviour in Matrigel but the cells did not attach or invade into the collagen matrix. This finding supports previous research which showed that SKOV3 does not attach or invade into collagen^14^ or form intraperitoneal tumours^15^.

The collagen invasion assay is more qualitatively informative than the Matrigel invasion assay as it is possible to visualise the morphology of the invading cells by taking a cross-section of the collagen matrix. TOV21G cells formed a thin layer of cells on top of the collagen, degraded the collagen or proliferated in a cavity and invaded as single cells with mesenchymal morphology. The single cells invaded outwards from the proliferative cell layer. This spindle-like morphology is characteristic of sarcomatous tumour cell invasion^16,17^ commonly associated with epithelial-mesenchymal transition (EMT)^18^. A2780 cells formed a thick proliferative cell layer on top of the collagen and invaded in small clumps of cells. This cluster invasion phenotype is characteristic of epithelial tumour cells^16^.

## Discussion

Previous studies investigating ovarian cancer cell line behaviour commonly use cell lines such as OVCAR3 and SKOV3 and focus on investigating how genetic manipulation or drug treatments can increase or decrease invasive and migratory behaviour. However, to our knowledge the endogenous behaviour of these cell lines stratified by histotype has not been carried out previously. We show that high-grade serous carcinoma-derived (HGS) cell lines behave differently from cell lines derived from non-serous carcinomas (NS) in four different behavioural assays. Our findings highlight the need for careful cell line selection in order that the data derived from these cell lines can be interpreted appropriately.

In general, the NS cell lines migrated more rapidly than HGS cell lines, which seems counter-intuitive as HGS tumours are typically more clinically aggressive, spread throughout the peritoneum and have a worse patient prognosis compared to NS tumours. Moreover, NS ovarian tumours commonly present at an early stage before metastasis has occurred. However, establishing cell lines from primary tumours *in vitro* is very difficult and many of the genetic mutations required to immortalise cell lines are found only in late-stage tumours, consistent with the observation that most cell lines have the potential to grow as metastases^19^. In keeping with this argument, TOV21G was derived from stage 3 clear cell carcinoma^20^; and SKOV3 and OAW42 were both derived from ascitic fluid (from post-chemotherapy patients)^21,22^, which is associated with higher stage disease. Given that NS tumours are normally diagnosed and treated prior to metastasis, the behaviour differences of these cell lines cannot therefore be used to replicate and understand the behaviour of primary NS tumours; however, they are useful for understanding the rare but more aggressive advanced stage NS disease.

It is important to highlight that although the HGS and NS cell lines showed different migration behaviour when analysed as distinct groups, the behaviours of individual cell lines did not segregate as clearly. A2780 is an NS cell line but behaved similarly to PEO1, PEO4 and FUOV1 which are HGS cell lines. Similarly, COV318 is classified genomically as a HGS cell line but behaves similarly to TOV21G and OVCAR8 which are NS cell lines. These findings highlight that, although these cell lines can be divided into different categories by their mutation profiles, their behaviours *in vitro* do not necessarily segregate the same way. Most importantly the cell lines do not all behave as expected based on their putative identity.

The link between cancer cell invasion and cell cycle arrest in G_0_/G_1_ is well documented^23,24^. Once a cell enters S phase it is committed to DNA replication, which is a very energy intensive process, making it unlikely that these cells can be involved in other cellular processes, such as invasion. The cell cycle findings provide evidence that the migration behaviour differences are not a result of proliferation. Furthermore, we found no systematic relationship between doubling time, estimated by cell count, and migration behaviour for the cell lines used in this study. For example, A2780, HACAT and TOV21G cells all had similar doubling times (14 hr, 15 hr and 18 hr respectively) but their migration behaviour was markedly different; A2780 migrated much more slowly than TOV21G and HACAT, failing to close the wound area after 60 hr. In contrast HACAT cells migrated quickly, closing the wound area in 33 hr but TOV21G cells took 60 hr despite a similar proliferation rate. These results are also in keeping with data derived from live imaging of invasion showing that cells in G_1_/G_0_ are more likely to invade and, if the same invasive cells cycle into S phase, they cease invasion until the cell cycle is complete^24^.

Similarly to migration, the finding that HGS cell lines are not invasive may seem unexpected. However, HGS tumours do not have to physically invade through tissue to seed metastatic tumours throughout the peritoneal cavity^8^. Even very large tumours only invade the superficial layers of the bowel and omentum and not the deeper layers^8^. It is rare to find HGS metastases to the bone or brain, which are common with other epithelial tumours such as breast and colon cancers which undergo several stages of intra-/extravasation before they seed metastases in other organs^8^. The behaviour of the NS cell lines is similarly counter-intuitive given that the disease is primarily non-metastatic. However, TOV21G was derived from the tumour of a patient diagnosed with stage 3 clear cell carcinoma^20^ and, anecdotally, patients with advanced stage NS tumours have a poor prognosis, similar to or even worse than those with HGS tumours, in keeping with the hypothesis that the behaviour of TOV21G replicates the rare advanced stage of the disease. Information on the origins of SKOV3 and OVCAR8 is limited^21,25^ but SKOV3 was derived from ascitic fluid rather than primary tumour^21,26,27^. Given that the formation of ascites is associated with advanced disease the invasive behaviour exhibited by SKOV3 also represents the behaviour of advanced stage disease^28^. HGS cells are characterised by a *TP53* mutation but OVCAR8, which is categorised as non-serous and behaves similarly to other NS cell lines, also has a *TP53* mutation (in addition to other mutations typically associated with non-serous tumours). It is therefore unlikely that, in these cell lines, the differences in the migration and invasion behaviours of HGS compared to NS cells are driven by *TP53* mutation as previously suggested by Ahn *et al*.^29^. Indeed, the most motile cell lines (including OVCAR8) have mutations in signalling pathways such as MAPK, PI3K and WNT^11^, which generally lead to overactivity in those pathways, and could explain the NS phenotype we observe. HGS cell lines characterised by only a *TP53* mutation would not have this phenotype and would be susceptible to genomic instability. In parallel, clinical evidence supports the idea that HGS tumour cells do not have to migrate quickly to seed metastasis as the disease develops undetected over several years.

The differences in invasive behaviour between HGS and NS cell lines used in this study are supported by *in vivo* tumourigenicity experiments. Hernandez *et al*.^30^ compared the ability of a different but overlapping panel of ovarian cancer cell lines to form tumours in nude mice over 6 weeks. They found that OVCAR8, SKOV3 and A2780 had high tumourigenicity when injected intraperitoneally, whereas OVCAR3, PEO1 and PEO4 had low tumourigenicity. Although the cell lines were not stratified by histotype in the *in vivo* study, the tumourigenic cell lines OVCAR8, SKOV3 and A2780 are derived from NS tumours and the non-tumourigenic cell lines OVCAR3, PEO1 and PEO4 are derived from HGS tumours.

Replicating the tumour microenvironment *in vitro* is challenging. The transwell invasion assay uses Matrigel, an extract of Engelbreth-Holm-Swarm (EHS) tumour which contains basement membrane components. Matrigel is commonly used in invasion assays but its suitability as an *in vitro* basement membrane substitute has been challenged^14,31^. Furthermore, the epithelial ovarian cancer microenvironment is composed of various different proteins and cell types. Collagen I is the most abundant structural protein within the ovarian extracellular matrix (ECM)^32^; therefore we used the collagen/fibroblast organotypic invasion assay to determine whether ovarian cancer cell lines behave differently when they encounter an ECM component more replicative of the *in vivo* environment.

The heterogeneity of the NS cell lines compared to the HGS cell lines may in part be explained by the fact that the NS category encompasses cell lines which were derived from clear cell and endometrioid carcinomas^20,21,33–35^. However, this heterogeneity cannot be explained by differences in tumour origin or molecular profile as both are associated with endometriosis and they have a similar genomic landscape. The differences in behaviours of NS cell lines in Matrigel and collagen highlights the importance of the substrate and may be related to the ECM context that the original cell line was derived from. Although the details in the original paper are sparse, A2780 was derived from untreated primary tumour material^34^. TOV21G was also derived from tumour material whereas SKOV3 was derived from ascites^20,21^. It is therefore interesting that TOV21G invades aggressively into both Matrigel and collagen, whereas SKOV3 shows invasion into Matrigel but not collagen; conversely A2780 invades aggressively into collagen but not Matrigel. Moreover, all the HGS cell lines apart from FUOV1 are derived from ascites^35–39^. Thus, overall, 2 of the 3 cell lines derived from primary tumours (TOV21G and A2780) attached and invaded into collagen but none of the cell lines derived from ascites exhibited this behaviour. This suggests that cell lines derived from the primary tumour have the ability to attach and invade into collagen whereas cell lines derived from ascites may not. This is consistent with the fact that tumour cells floating in ascitic fluid are able to survive and grow in suspension, without attachment to, or invasion into, a solid matrix.

Cell lines are vital as a first step to understanding molecular and behavioural changes that result in tumour development and progression. However, the current approach of using cell lines to investigate ovarian cancer, without stratifying by histotypes, is hindering the advancement of the field. Our data provide evidence that ovarian cancer cell line behaviour correlates to some extent with tumour histotype and hence origin. However, the variability between cell lines indicates that behavioural characterisation should be taken into account, in addition to tumour histotype, origin and molecular profile, when interpreting the results of *in vitro* experiments using these cell lines.

Taken together these findings provide a basis for the selection of relevant ovarian cancer cell lines to investigate the differences between HGS and NS tumours *in vitro*. Representative *in vitro* models will help overcome the current gap in the translation of laboratory research to novel therapies for ovarian cancer patients. Future research should focus on using appropriately selected cell lines for the development of *in vitro* models to understand the molecular and behavioural differences between ovarian tumour types.

## Materials and Methods

All reagents were purchased from Thermo Fisher Scientific (Renfrew, UK) unless otherwise stated.

### Cell culture

Human epithelial ovarian adenocarcinoma cell line SKOV3 was acquired from ATCC. Human epithelial ovarian adenocarcinoma cell lines NIH: OVCAR-3 (OVCAR3), OVCAR8, PEO1, PEO4, COV318 and FUOV1 were kindly supplied by David Melton from the University of Edinburgh. Human epithelial ovarian adenocarcinoma cell lines A2780, TOV21G and OAW42 were kindly supplied by Charlie Gourley from the University of Edinburgh. Human keratinocyte cell line HACAT was kindly supplied by Lana Woolford from the University of Edinburgh. All cell lines were authenticated using STR profiling (Public Health England). Red fluorescent protein telomerase reverse transcriptase (TERT) immortalised fibroblasts (RTIF) generated from skin punch biopsies were a kind gift of Andrew Campbell (Beatson Institute, Glasgow). All cell lines were maintained in a humidified incubator at 37 °C with 5% CO_2_. OVCAR3, SKOV3, OVCAR8, PEO1, PEO4, COV318, FUOV1, A2780, TOV21G and OAW42 were cultured in RPMI Medium 1640 (1 × ) supplemented with 10% fetal calf serum (FCS), 1X non-essential amino acids (Sigma Aldrich, Irvine, UK), 100 µL/mL (v/v) penicillin-streptomycin, 1 mM sodium pyruvate (Sigma Aldrich) and 1 µL/mL bovine insulin (Sigma Aldrich). All ovarian cancer cell lines were cultured in the same medium to control for biases due to varied levels of growth factors or different serum levels, in keeping with the approach of Beaufort *et al*.^12^. All of the lines had been acclimatised to this medium for at least 3 months prior to experimentation. HACAT was cultured in DMEM (1×) + GlutaMAX supplemented with 10% FCS, 100 µL/mL (v/v) penicillin-streptomycin. Fibroblasts were cultured in DMEM (1×) +GlutaMAX supplemented with 10% FCS. Cell lines were routinely tested for mycoplasma contamination and all cell experiments were carried out in sterile conditions. Cell lines were passaged using 0.05% trypsin-EDTA (1×). All experiments were carried out with cell lines no more than 10 passages apart to ensure biological repeats and limit the mutational effect of passaging. Cell lines were not synchronised prior to experimentation.

### Wound healing assay

For each cell line, 100 µL of a 5 × 10^6^ cells/mL cell suspension (5 × 10^5^ cells) were seeded into every well of an ImageLock™ 96 well plate (Essen Bioscience, Hertfordshire, UK). The plate was then incubated at 37 °C, 5% CO_2_ for 4 hours (hr). After the incubation period, the 96 well WoundMaker™ (Essen Bioscience) was used to simultaneously create wounds in all wells. The plate was then washed twice with culture media (100 µL per well; media specific to cell line used) to remove any cell debris. 100 µL of normal cell culture media was then added to each well and the plate was placed inside the IncuCyte ZOOM® incubator system (Essen Bioscience). Plates were imaged every 3 hr for 60 hr. The percentage area covered by cells inside the wound area relative to the percentage area covered by cells outside of the wound area (relative wound density) at every time point was analysed. 3 technical repeats were carried out for each cell line and this procedure was repeated 3 times to ensure 3 biological repeats.

### Flow cytometry

For each cell line, 5 × 10^5^ cells were transferred to a fluorescence-activated cell sorting (FACS) tube and the content centrifuged for 5 minutes (min) at 400xg. To fix the cells, the supernatant was discarded and the pellet resuspended in 300 μL PBS supplemented with 50% FCS. 900 μL of ice-cold 70% ethanol was then slowly added whilst gently tube vortexing. The samples were stored at 4 °C. On the day of analysis, 3 mL PBS was added to the samples and the content centrifuged for 5 min at 400xg. The supernatant was discarded and the pellet re-suspended in 300 μL PBS supplemented with 0.1 μL/mL DAPI, 0.1% Triton x100. The samples were then stored for 1 hr in the dark and analysed using flow cytometry (BD LSRFortessa X-20). This procedure was carried out with 3 biological repeats per cell line.

### Inverted invasion assay

This method is a modified version of Hennigan *et al*.^40^. Matrigel (Corning, New York, USA) was diluted in a 1:1 ratio with 1% serum culture media. Then 100 μL of the dilute Matrigel was added into the centre of each Transwell (Corning) and the plate was incubated for approximately 1 hr at 37 °C, 5% CO_2_. During this time, a cell suspension of 5 × 10^5^ cells/mL was prepared in normal cell culture media for each cell line. Once the Matrigel had set, the transwells were inverted and placed onto the lid of the 24-well plate. Next 100 μL of the cell suspension (5 × 10^4^ cells) was pipetted onto the underside of the filter. The transwells were then carefully covered with the base of the plate and placed (inverted) in an incubator at 37 °C, 5% CO_2_ for 3 hr to allow the cells to attach to the transwell. After the incubation period, the plate was turned the right way up and each transwell dipped into 1 mL 1% serum media to wash; this was repeated 3 times. The transwell was left in the final wash media and 100 μL normal cell culture media was added to the top of the transwell (on top of the Matrigel). The plate was then incubated at 37 °C, 5% CO_2_ for 5 days. On the 5^th^ day SYTO™9 green fluorescent nucleic acid stain was diluted 1/2000 into 1% serum media in a falcon tube and vortexed. Next 0.5 mL of this solution was added to the wells of a 24-well plate. The plate was removed from the incubator and a transwell was added to each of the wells with SYTO™9 media. Then 0.5 mL of the SYTO™9/media solution was pipetted onto the top of each transwell to submerge the gel in staining solution. The plate was then incubated for at least 1 hour at 37^o^C with 5% CO_2_ and imaged immediately afterwards.

### Imaging and quantification of inverse invasion assays

Transwells were placed on a thin coverslip and images were acquired on a Nikon Confocal A1R confocal microscope using a 20X NA 0.75 objective. The microscope is equipped with a 405 nm diode, Argon laser and 561 and 648 nm laser lines and four photomultiplier tubes. Data were acquired using NIS Elements AR software (Nikon Instruments Europe, Netherlands). Images were scanned at 1x zoom, 0.25 speed and 1x line average. Optical sections were scanned at 15 μm intervals (Z-steps) moving up from the underside of the filter into the Matrigel. The percentage area covered by cells in each Z-step image was quantified in Image J using the thresholding feature. For quantification purposes, only cells in the Z-step 45μm or above were considered invasive as described previously^13^. Three separate assays were carried out per cell line giving 3 biological repeats with four Z-series taken for each sample per assay.

### Collagen/fibroblast organotypic invasion assay

Collagen I was prepared from rat tails as described by Timpson *et al*.^41^. A confluent T75 flask of fibroblasts was passaged and a cell suspension of 1 × 10^6^ cells prepared. The fibroblasts were then centrifuged at 400xg for 5 min. During this time, the collagen mix was prepared and all components kept on ice to ensure the collagen did not set; 3 mL MEM (10 × ) was added to 25 mL rat tail collagen I (approximately 2.6 mg/mL). Then 0.25 M NaOH (in-house) was added dropwise whilst gently mixing until the collagen mix turned orange but not pink (approximately 2.5 mL NaOH). The fibroblasts were resuspended in 3 mL FCS, added to the collagen solution and gently mixed. 2.5 mL of the collagen/fibroblast mixture was then quickly added to each well of two 6-well plates avoiding any bubbles. The plates were incubated at 37 °C, 5% CO_2_ for 10 min to allow the mixture to set. The collagen/fibroblast matrix was then detached from the sides of the wells and 1 mL of fibroblast culture media carefully added on top of the matrix. The plates were incubated at 37 °C, 5% CO_2_ for approximately 8 days to allow the collagen/fibroblast matrix to contract to 1.5 cm diameter.

Once the collagen/fibroblast matrices had contracted, blunt forceps were used to gently move them to a 24-well plate. All cell lines were then split and resuspended in normal growth media at approximately 8 × 10^4^/mL. 1 mL of the cell suspension was then seeded on top of the matrix. The 24-well plate was then incubated at 37 °C with 5% CO_2_ for 5 days to allow cells to grow confluent.

Tripods were made out of stainless steel grids and autoclaved prior to use. The sterile grids were placed into a 6 cm dish and normal cell culture media added to a level just above the grid. Using blunt forceps the collagen/fibroblast matrix was transferred to the grid and the media gently aspirated so that the bottom of the matrix is in contact with media but not submerged. This creates an air/liquid interface to facilitate invasion. Dishes were incubated at 37 °C with 5% CO_2_ for 10 days to allow the cells to invade. After 10 days the matrix was removed from the grid and cut in half using a scalpel. The collagen matrix was then added to a falcon tube with 5 mL 4% (w/v) paraformaldehyde to fix overnight.

### Histopathology

The fixed collagen/fibroblast matrix was embedded in wax, sectioned and stained with haematoxylin and eosin (H&E) by the CRUK Edinburgh Centre Pathology & Phenomics Lab. Stained sections were imaged on the Nanozoomer XR model, data captured using NDP scan v 3.1. 40x magnification.

### Statistical analysis

All statistical analysis was carried out in SPSS and graphs created in GraphPad Prism unless otherwise stated. The Kolmogorov-Smirnov (KS) test was used to determine the normality of the data. For normally distributed data (KS: p > 0.05), parametric tests such as the independent samples t-test were used. For non-normally distributed data (KS: p < 0.05), the non-parametric Mann-Whitney U test was used. Significance was set at p < 0.05 for all statistical analyses. Wound healing data were analysed using two-way repeated measures ANOVA (GraphPad Prism); the normality of the data was assumed and limitations of this are acknowledged.

## Data Availability

All data generated or analysed during this study are included in this published article.
